# Microstructural and Microvascular Phenotype of Sarcomere Mutation Carriers and Overt Hypertrophic Cardiomyopathy

**DOI:** 10.1161/CIRCULATIONAHA.123.063835

**Published:** 2023-07-18

**Authors:** George Joy, Christopher I. Kelly, Matthew Webber, Iain Pierce, Irvin Teh, Louise McGrath, Paula Velazquez, Rebecca K. Hughes, Huafrin Kotwal, Arka Das, Fiona Chan, Athanasios Bakalakos, Massimiliano Lorenzini, Konstantinos Savvatis, Saidi A. Mohiddin, Peter W. Macfarlane, Michele Orini, Charlotte Manisty, Peter Kellman, Rhodri H. Davies, Pier D. Lambiase, Christopher Nguyen, Jurgen E. Schneider, Maite Tome, Gabriella Captur, Erica Dall’Armellina, James C. Moon, Luis R. Lopes

**Affiliations:** Barts Heart Centre, Barts Health NHS Trust, London, UK (G.J., I.P., P.V., R.K.H., H.K., A.B., M.L., K.S., S.A.M., M.O., C.M., R.H.D., P.D.L., J.C.M., L.R.L.).; Institute of Cardiovascular Science (G.J.. M.W., I.P., R.K.H., F.C., A.B., M.L., K.S., M.O., C.M., R.H.D., P.D.L., G.C., J.C.M., L.R.L.), University College London, UK.; Medical Research Council Unit for Lifelong Health and Ageing (M.W., I.P., F.C., R.H.D., G.C.), University College London, UK.; Biomedical Imaging Sciences Department, Leeds Institute of Cardiovascular and Metabolic Medicine, University of Leeds, UK (C.I.L., I.T., A.D., J.E.S., E.D.).; Centre for Inherited Heart Muscle Conditions, Department of Cardiology, Royal Free London NHS Foundation Trust, UK (M.W., F.C., G.C.).; Imaging Department, Royal Brompton & Harefield Hospitals, London, UK (L.M.).; Cardiology Clinical and Academic Group, St. Georges University of London and St. Georges University Hospitals NHS Foundation Trust, UK (P.V., M.T.).; William Harvey Research Institute, Queen Mary University London, UK (K.S., S.A.M.).; Electrocardiology Section, School of Health and Wellbeing, University of Glasgow, UK (P.W.M.).; National Heart, Lung, and Blood Institute, National Institutes of Health, DHHS, Bethesda, MD (P.K.).; Cardiovascular Innovation Research Centre, HVTI, Cleveland Clinic, OH (C.N.).

**Keywords:** cardiomyopathy, hypertrophic, diffusion tensor imaging, magnetic resonance imaging, microcirculation, perfusion, sarcomeres

## Abstract

**BACKGROUND:**

In hypertrophic cardiomyopathy (HCM), myocyte disarray and microvascular disease (MVD) have been implicated in adverse events, and recent evidence suggests that these may occur early. As novel therapy provides promise for disease modification, detection of phenotype development is an emerging priority. To evaluate their utility as early and disease-specific biomarkers, we measured myocardial microstructure and MVD in 3 HCM groups—overt, either genotype-positive (G+LVH+) or genotype-negative (G−LVH+), and subclinical (G+LVH−) HCM—exploring relationships with electrical changes and genetic substrate.

**METHODS:**

This was a multicenter collaboration to study 206 subjects: 101 patients with overt HCM (51 G+LVH+ and 50 G−LVH+), 77 patients with G+LVH−, and 28 matched healthy volunteers. All underwent 12-lead ECG, quantitative perfusion cardiac magnetic resonance imaging (measuring myocardial blood flow, myocardial perfusion reserve, and perfusion defects), and cardiac diffusion tensor imaging measuring fractional anisotropy (lower values expected with more disarray), mean diffusivity (reflecting myocyte packing/interstitial expansion), and second eigenvector angle (measuring sheetlet orientation).

**RESULTS:**

Compared with healthy volunteers, patients with overt HCM had evidence of altered microstructure (lower fractional anisotropy, higher mean diffusivity, and higher second eigenvector angle; all *P*<0.001) and MVD (lower stress myocardial blood flow and myocardial perfusion reserve; both *P*<0.001). Patients with G−LVH+ were similar to those with G+LVH+ but had elevated second eigenvector angle (*P*<0.001 after adjustment for left ventricular hypertrophy and fibrosis). In overt disease, perfusion defects were found in all G+ but not all G− patients (100% [51/51] versus 82% [41/50]; *P*=0.001). Patients with G+LVH− compared with healthy volunteers similarly had altered microstructure, although to a lesser extent (all diffusion tensor imaging parameters; *P*<0.001), and MVD (reduced stress myocardial blood flow [*P*=0.015] with perfusion defects in 28% versus 0 healthy volunteers [*P*=0.002]). Disarray and MVD were independently associated with pathological electrocardiographic abnormalities in both overt and subclinical disease after adjustment for fibrosis and left ventricular hypertrophy (overt: fractional anisotropy: odds ratio for an abnormal ECG, 3.3, *P*=0.01; stress myocardial blood flow: odds ratio, 2.8, *P*=0.015; subclinical: fractional anisotropy odds ratio, 4.0, *P*=0.001; myocardial perfusion reserve odds ratio, 2.2, *P*=0.049).

**CONCLUSIONS:**

Microstructural alteration and MVD occur in overt HCM and are different in G+ and G− patients. Both also occur in the absence of hypertrophy in sarcomeric mutation carriers, in whom changes are associated with electrocardiographic abnormalities. Measurable changes in myocardial microstructure and microvascular function are early-phenotype biomarkers in the emerging era of disease-modifying therapy.

**Clinical Perspective**

**What Is New?**

- With the use of diffusion tensor imaging in hypertrophic cardiomyopathy, evidence of myocardial disarray is detectable in pathogenic sarcomere mutation carriers even in the absence of hypertrophy.
- Diffusion tensor imaging parameters suggestive of myocardial disarray are related to electrocardiographic abnormalities in subclinical and overt hypertrophic cardiomyopathy.
- In overt disease, the presence versus absence of sarcomeric mutation has differing effects on microstructure and microvasculature.

**What Are the Clinical Implications?**

- Diffusion tensor imaging may discriminate pathogenic sarcomere mutations from health and mutation status in overt disease.
- Disarray and ischemia are associated with stage of phenotype evolution.

**Editorial, see p 819**

Hypertrophic cardiomyopathy (HCM) is a leading cause of heart failure and sudden death.^1^ Characterized clinically by hypertrophy, HCM has considerable heterogeneity of presentation and outcomes.^2,3^ Cardiac function, extreme hypertrophy, and extent of fibrosis are known to be adverse markers, but these may be late in phenotype development and may only modestly predict adverse events.^2–5^ Histologically, HCM is characterized by myocyte hypertrophy and fibrosis but also by 2 other features: small-vessel disease and disarray.^5,6^ It is thought that these may be important in both phenotype development, from subclinical (in the absence of hypertrophy) to overt disease, and risk; but until recently, these have been hard to measure clinically, and no study has measured them concurrently.^7–9^ Novel advances permit quantification of microstructural indices indicative of disarray and microvascular disease (MVD), bypassing the need for tissue specimens or ionizing radiation.^10,11^

Cardiac diffusion tensor imaging (cDTI) measures the diffusion of water within an imaging voxel, thereby characterizing the myocardial microstructural environment. HCM is characterized by: (1) low fractional anisotropy (FA), a measure of the directional variability of water diffusion, with low values suggestive of myocyte disarray and collagen deposition; (2) high mean diffusivity (MD), measuring the magnitude of diffusion, thought to reflect myocyte packing, with high values reflecting increased interstitial fibrosis and changes in intracellular and extracellular volume; and (3) elevated absolute second eigenvector angle (|E2A|), reflecting sheetlet orientation (functional units of myocytes that dynamically reorientate throughout the cardiac cycle to facilitate wall thickening). Sheetlets conform to hypercontracted states (elevated |E2A|) in systole with failure to reorientate in diastole, implying roles in diastolic failure in HCM and increased cardiomyocyte tension^12–15^ (Figure 1).

**Figure 1.**
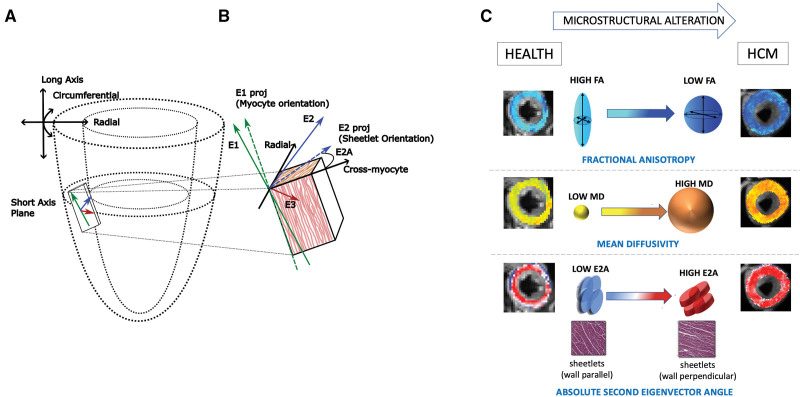
**Cardiac diffusion tensor imaging. A**, Representation of the short-axis imaging plane with a magnified imaging voxel containing ≈50 000 myocytes. Diffusion tensor imaging measures magnitudes (eigenvalues λ1, λ2, and λ3) and direction (eigenvectors E1, E2, and E3) of water diffusion. **B**, Three arrows (green, blue, and red) representing the 3-dimensional directions of diffusion: the 3 principal eigenvectors. E1 (green) is the direction of maximum diffusivity orientated along the myocyte long axis. Myocytes are organized into sheetlets, functional units of myocytes that dynamically reorientate to facilitate wall thickening, reflected by the E2 (blue) angle against the cross-myocyte plane (right angles to E1 projection on the wall tangent plane). **C**, Sheetlets angled parallel to the wall tangent have a low absolute second eigenvector angle (|E2A|), and sheetlets positioned perpendicular to the wall tangent have a high |E2A|, signifying a more contracted sheetlet configuration. If myocytes were perfectly randomly orientated (isotropic diffusion), fractional anisotropy (FA) would be 0; hence, myocyte disarray causes lower fractional anisotropy (FA) values. Mean diffusivity (MD) is the mean of the eigenvalues, with higher values representing a greater magnitude of diffusion. MD is thought to be sensitive to myocyte packing and intracellular and extracellular volume shifts. HCM indicates hypertrophic cardiomyopathy. Adapted from Ariga et al^22^ (Copyright © 2019 Elsevier) and Ferreira et al^14^ (Copyright © 2014 Ferreira et al, licensee BioMed Central Ltd), Nielles-Vallespin et al^15^ (Copyright © 2017 Elsevier), and Das et al^13^ (Copyright © 2021 Radiological Society of America) with permission.

Patients with overt HCM without a detectable sarcomeric mutation, called genotype-negative (G−LVH+), represent approximately half of patients and are characterized by fewer events (heart failure or ventricular arrhythmia), less fibrosis (lower extracellular volume [ECV] or less scar), and differing morphology (more apical HCM or more isolated basal septal left ventricular [LV] hypertrophy [LVH]).^1,16,17^ Individuals with pathogenic sarcomeric variants without hypertrophy (G+LVH−) identified on cascade genetic screening have architectural and functional abnormalities (crypts, trabeculation, hyperdynamic function, impaired diastolic function, and myocardial mechanics). Furthermore, G+LVH- with abnormal electrocardiographic findings have a 4-fold increased risk of progression to overt disease.^18–20^

As novel sarcomere modulators provide promise for disease modification, early markers of disease are a rapidly emerging research priority.^21^ Analyzing the effect of sarcomeric mutation on phenotype development will enhance precision medicine in the future.^1,5,22^ We aimed to investigate the prevalence, extent, and interrelationships of microstructural alteration and MVD in both overt and subclinical HCM. We then studied the associations between these biomarkers and pathological electrocardiographic findings, nonsustained ventricular tachycardia (NSVT), and genetic substrate (genotype positive [G+LVH+] and genotype negative [G−LVH+]).

## METHODS

### Study Population

The data that support the findings of this study are available from the corresponding author on reasonable request. We formed an academic collaboration consisting of 3 recruiting tertiary referral centers (Barts Heart Centre, St. George’s University Hospital, and Royal Free Hospital, London, UK) and a technological collaboration of 2 US sites and 2 UK sites to deploy advanced cardiac magnetic resonance imaging (CMR) techniques: cDTI: Cleveland Clinic (Cleveland, OH) and Leeds Institute of Cardiovascular and Metabolic Medicine (Leeds, UK); and quantitative perfusion mapping: National Institutes of Health (Bethesda, MD) and Barts Heart Centre. Participants with subclinical or overt HCM who were 18 to 76 years of age were prospectively and consecutively recruited from databases of genotyped patients. Ethics approval for the study was given by the research ethics committee (IRAS 227168). All subjects gave informed consent for all study procedures. Pathogenicity for detected variants was assessed with American College of Medical Genetics criteria.^23^ Inclusion criteria were as follows: (1) Overt HCM (G+LVH+ and G−LVH+) was diagnosed according to American Heart Association and European Society of Cardiology guidelines (increased LV wall thickness that is not solely explained by abnormal loading conditions; maximum wall thickness [MWT] ≥15 mm in any cardiac segment by any imaging modality or ≥13 mm in patients with a first-degree relative with confirmed HCM).^2,3^ (2) Patients with subclinical HCM (G+LVH−) had pathogenic/likely pathogenic variants confirmed on cascade screening but without hypertrophy (defined as MWT <13 mm; 2 patients were found to have hypertrophy on their research CMR and were therefore transferred to the G+LVH+ group). (3) Healthy volunteers (HVs) with no relevant medical history or risk factors for coronary disease were prospectively matched for age, sex, and ethnicity to the subclinical HCM (G+LVH−) cohort under the same ethics protocol. A separate age-, sex-, and ethnicity-matched cohort of HVs was compared with the overt HCM group to assess whether any diffusion tensor imaging (DTI) changes were attributable to demographics variables (n=24; 11 overlapped with the younger HV group). HVs were not genotyped and were not relatives of those with HCM. To optimize conditions for cDTI and to reduce the risk of confounding, those with known obesity (body mass index ≥30 kg/m^2^) and poorly controlled hypertension (poor blood pressure control despite 2 antihypertensives) were excluded. Presence of LV outflow obstruction and gradients was collected from clinical echocardiographic data at the time of recruitment.

Other exclusion criteria were as follows: claustrophobia, unwillingness to consent, known coronary disease or significant pretest probability for coronary disease without previous coronary imaging, contraindications to adenosine, pulmonary hypertension, previous alcohol septal ablation or myectomy, previous cardiovascular surgery, congenital disease, and implantable cardiac devices.

### CMR Image Acquisition and Analysis

All scans were acquired on a single 3-T Prisma (Siemens Healthineers, AG, Erlangen, Germany). Standard long- and short-axis cine imaging was performed, with segmentation for ventricular dimensions and function performed with fully automated algorithms that exceed human precision.^24,25^ T1 mapping before and after contrast was performed with Modified Look-Locker Inversion Recovery (MOLLI 5s[3s]3s) with automated contouring using Circle CVI42 software (version 5.14.1) and manual adjustment when required for septal ECV (using same-day hematocrit) in line with recommendations.^26^ Fully quantitative vasodilator stress perfusion was performed with a validated dual sequence approach as previously described.^10^ In brief, adenosine was given intravenously at 140 to 210 µg·kg^−1^·min^−1^ for a minimum of 4 minutes, until a minimum heart rate increase of 15 bpm and symptoms suggestive of an adequate physiological stress response. Gadolinium-based contrast (Dotarem, Gadoteric Acid, Guerbet, UK) was then administered intravenously at 0.05 mmol/kg. The same was repeated for rest imaging, which was acquired at a minimum of 7 minutes after adenosine. Automated in-line adenosine stress perfusion maps were acquired to obtain stress and rest myocardial blood flow (MBF; in milliliters per gram per minute), myocardial perfusion reserve (MPR; ratio of stress and rest MBF), and visual perfusion defects (defined according to clinically recommended task force criteria^27^) assessed separately by 2 experienced operators (G.J. and J.C.M.) from conventional images and perfusion maps.^28^

A second-order motion–compensated single-shot spin-echo echo-planar imaging DTI sequence was performed for 3 short-axis slices at peak systole following previously described protocols.^12,13^ This was free breathing without respiratory navigation, has been validated both ex vivo and in vivo, and has been demonstrated to detect microstructural changes in HCM.^12,13,29^ cDTI analysis was performed according to previously described protocols; in brief, data processing was performed with custom-built Matlab software (MathWorks), with each image quality-controlled for misregistration or artifacts. Average magnitude images were generated from registered data by averaging cross-repetitions, and diffusion tensors were calculated. Contouring was performed on cDTI maps, with tensor eigenvalues, MD, FA, and absolute |E2A| obtained globally and segmentally. Segments containing artifacts were omitted from analysis.^12,13^ Late gadolinium enhancement (LGE) imaging was performed 5 minutes after administration of gadolinium-based contrast agent for pixelwise quantification of focal fibrosis (defined as signal intensity 5 SDs from the mean of a remote region of interest and expressed henceforth as absolute grams). Circle CVI 42 (version 5.14.1) was used for LGE map analysis with automated contouring of endocardial and epicardial borders and manual adjustment when required.

### Digital Electrocardiographic Analysis

A same-day standard resting digital 12-lead ECG was performed with a Mindray Beneheart R3 electrocardiograph (Shenzhen Mindray Bio-Medical Electronics Co), and automated analysis was performed by a core laboratory to identify abnormalities associated with disease progression: abnormal Q waves (defined as present in ≥2 contiguous leads and minimum amplitude of 0.3 mV, ≥25% of the subsequent R wave, or duration >40 milliseconds), Sokolow-Lyon index (SV1+RV5/6>3.5 mV) or Cornell criteria (RaVL+SV3>2.8 mV in men and >2.0 mV in women), and repolarization abnormalities (defined as T-wave inversion in ≥0.1 mV in ≥2 contiguous leads or ST-segment depression ≥0.1 mV in ≥2 contiguous leads).^20,30^ For overt HCM, Holter recordings were available in 99% of patients (101/102) within 6 months of the CMR, with NSVT defined as 3 beats ≥120 bpm occurring on Holter monitoring.

### Statistical Analysis

Statistical analysis was performed with SPSS (IBM SPSS Statistics, version 28). Continuous variables were described as mean±SD for normally distributed variables and compared with independent-samples *t* test. For nonnormally distributed variables (tested with the Shapiro-Wilk test), median and interquartile range with equivalent comparison tests were used. Categorical/binomial variables were expressed as absolute values and percentages and compared with χ^2^ or Fisher exact test as appropriate. Univariable and multivariable linear regression models were used to determine associations of diffusion tensor, perfusion, fibrosis, and hypertrophy parameters. Between-group comparisons involving DTI were adjusted for age (see later discussion), fibrosis, and hypertrophy (septal ECV, LGE mass, and MWT) and expressed as standardized β coefficients and 95% CIs. A variance inflation factor <3 excluded multicollinearity. Logistic regression models with inclusion of fibrosis (ECV and LGE) and hypertrophy (MWT) variables as covariates were used to test for odds ratios (ORs) for independent risk of electrocardiographic abnormalities and NSVT. Continuous predictors were normalized before entering the logistic regression, and ORs are expressed per 1-SD increase in MD and |E2A| and 1-SD decrease for FA, stress MBF, and MPR. A 2-sided value of *P*<0.05 was considered significant. Multiple testing correction with Bonferroni was done for the primary end points (perfusion and DTI in patients with G+LVH− versus HVs and G+LVH+ versus G−LVH+) in a table-by-table basis (adjusted *P* threshold=0.05/[number of parameters analyzed multiplied by number of comparisons]). Age, sex, ethnicity, and body mass index were tested for associations with DTI parameters (Supplemental Results) in pooled HVs (n=41), subclinical HCM, and overt HCM. MD was found to be associated with age in overt HCM; therefore, age was included in all between-group regression analyses involving DTI. An exploratory analysis was performed to determine whether any gene-specific differences could be detected (Supplemental Results).

## RESULTS

### Clinical Characteristics

Overall, 206 participants were studied: 101 with overt disease (51 G+LVH+ and 50 G−LVH+), 77 with subclinical HCM (G+LVH−), and 28 HVs. Of 77 with G+LVH−, pathogenic/likely pathogenic variants were 60% (46) *MYBPC3*, 23% (18) *MYH7*, 6% (5) *TNNI3*, 6% (5) *TNNT2*, 1% (1) *MYL2*, 1% (1) *TPM1*, and 1% (1) *CSRP3*. Of 51 with G+LVH+, pathogenic/likely pathogenic variants were 55% (28) *MYBPC3*, 25% (13) *MYH7*, 8% (4) *TNNI3*, 8% (4) *TNNT2*, 2% (1) *CSRP3*, and 2% (1) *TNNC1* (Table S3). Demographics and conventional CMR parameters are summarized in Table 1. Comparisons between overt HCM (all LVH+; n=101) and HVs are provided in Table S1. To examine the effect of LVH on parameters in those with pathogenic/likely pathogenic sarcomere variants, comparisons between G+LVH− and G+LVH+ are included in Table S2. Of the overt HCM cohort, 23% (23) had a peak LV outflow tract gradient ≥30 mm Hg; these patients’ average gradient at rest was 27 mm Hg (18–60 mm Hg) and on provocation was 56 mm Hg (38–85 mm Hg).

**Table 1.**
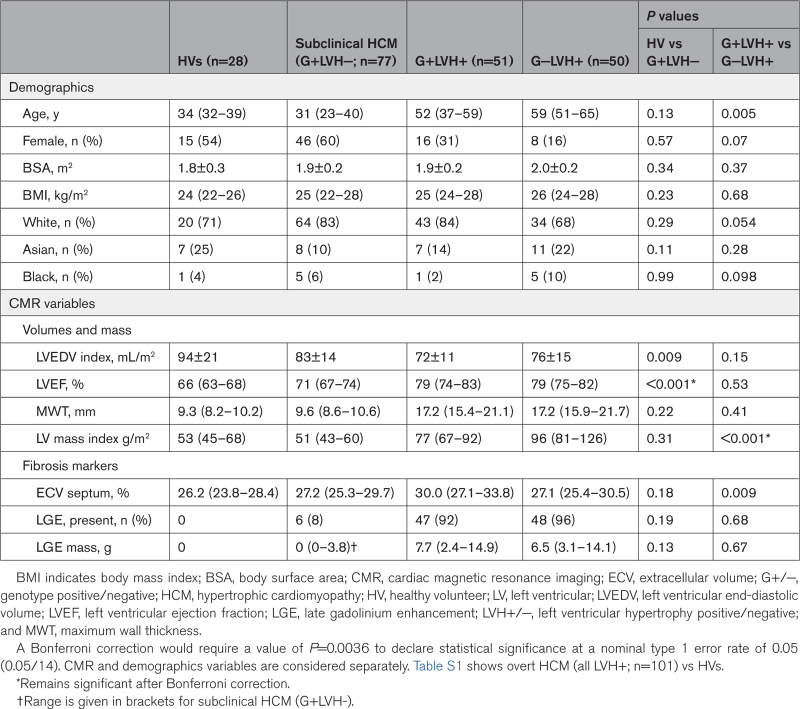
Demographics and CMR Variables Across the 4 Groups

#### Overt HCM: All LVH+ Versus HVs

Overt HCM was characterized by higher ejection fraction and more severe markers of hypertrophy (higher MWT and higher LV mass) and fibrosis (higher ECV and higher burden of LGE; Table S1).

#### Overt HCM: G+LVH+ Versus G−LVH+

There were no differences in ejection fraction and MWT. Patients with G−LVH+ had higher LV mass. G−LVH+ had similar LGE burden and lower ECV (but not after correction for multiple comparisons; Table 1).

#### Subclinical HCM: G+LVH− Versus HVs

Compared with HVs, patients with G+LVH− had a lower indexed end-diastolic volume, higher ejection fraction, but no difference in markers of hypertrophy (MWT or LV mass) and no difference in markers of fibrosis (ECV and LGE; Table 1).

### Myocardial Perfusion

#### Overt HCM (All LVH+) Versus HVs

Patients with HCM had evidence of MVD (lower stress MBF and MPR) with a higher prevalence of perfusion defects (91% [92/101] versus 0%; *P*<0.001; Table S1).

#### Overt HCM: G+LVH+ Versus G−LVH+

**Figure 2.**
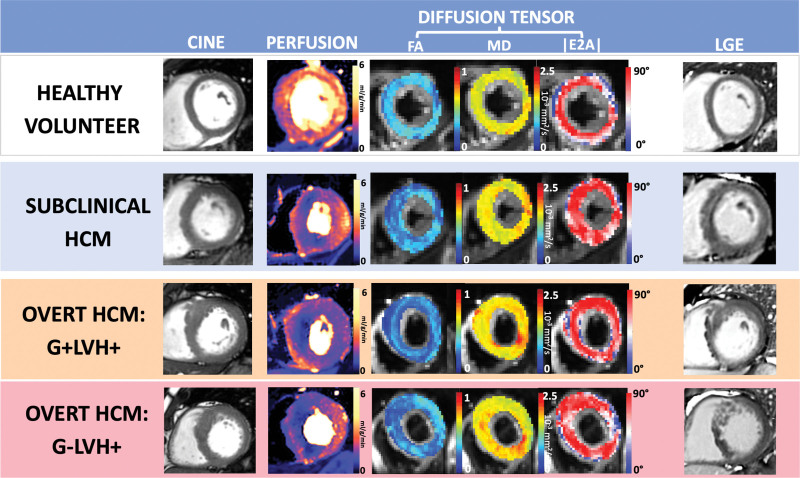
**Abnormalities in perfusion and diffusion tensor parameters (low FA, high MD, and high |E2A|) occurring in the absence of hypertrophy in subclinical HCM (G+LVH−) and more severely in overt disease (G+LVH+ and G−LVH+).** |E2A| indicates second eigenvector angle; FA, fractional anisotropy; G+/−, genotype positive/negative; LGE, late gadolinium enhancement; LVH+/−, left ventricular hypertrophy positive/negative; and MD, mean diffusivity.

**Figure 3.**
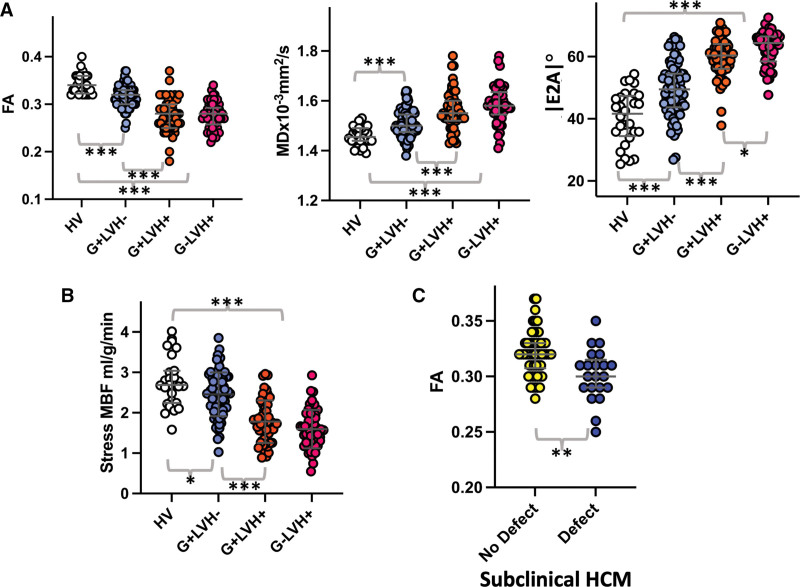
**Diffusion tensor and quantitative perfusion CMR parameter alterations in phenotype development. A**, Diffusion tensor parameter changes are detectable in subclinical (G+LVH−) hypertrophic cardiomyopathy (HCM) and measure more severely in overt disease. Genotype-negative HCM (G−LVH+) is characterized by elevated second eigenvector angle (|E2A|) compared with genotype-positive HCM (G+LVH+). **B**, Stress myocardial blood flow (MBF), reflecting microvascular disease, is reduced in subclinical HCM and more severely in overt disease. **C**, Subclinical HCM with perfusion defects had lower fractional anisotropy (FA; suggestive of more disarray) compared with subclinical HCM without perfusion defects. |E2A| indicates second eigenvector angle; HV, healthy volunteer; LVH, left ventricular hypertrophy; MD, mean diffusivity; and MPR, myocardial perfusion reserve. *P<0.05. **P<0.01. ***P<0.001.

There was no difference in global perfusion parameters (stress MBF or MPR), but all 51 G+LVH+ patients had perfusion defects compared with 82% (41/50) G−LVH+ patients (*P*=0.001; Table 2; Figure 2; Figure 3B).

**Table 2.**
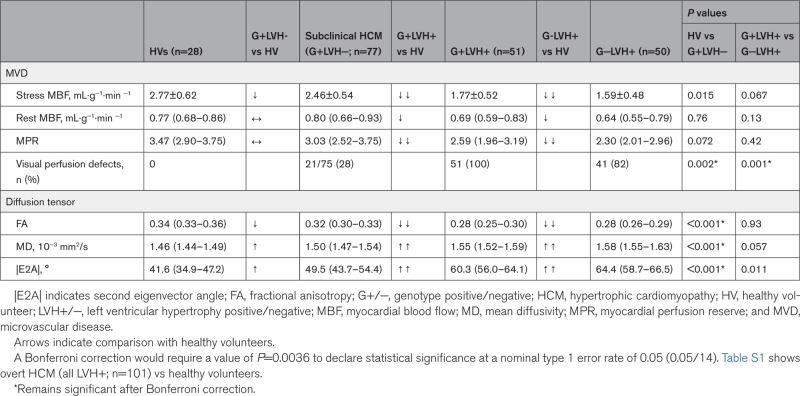
Diffusion Tensor and Quantitative Perfusion Parameters

#### Subclinical HCM (G+LVH−) Versus HVs

Patients with G+LVH− had reduced stress MBF but not after correction for multiple comparisons (Table 2; Figure 2; Figure 3B). Patients with subclinical HCM had a higher prevalence of perfusion defects (28% versus 0%; *P*=0.002) but no difference in MPR (Figure S1).

### Microstructural Indices Measured by DTI

Associations of DTI parameters, fibrosis, and MWT are presented in Supplemental Results.

#### Overt HCM (All LVH+) Versus HVs

Compared with HVs, patients with overt HCM had evidence of microstructural alteration: lower FA, suggestive of disarray and higher MD, and higher |E2A| suggestive of a more hypercontracted sheetlet configuration (Table S1). Differences remained after adjustment for fibrosis and hypertrophy (FA: β=−0.52 [95% CI, −0.32 to −0.68], *P*<0.001; MD: β=0.26 [95% CI, 0.05–0.45], *P*<0.015; |E2A|: β=0.64 [95% CI, 0.48–0.80], *P*<0.001). Results relating to DTI were similar compared with age-, sex-, and ethnicity-matched HVs (Supplemental Results).

#### Overt HCM: G+LVH+ Versus G−LVH+

Although MD and FA were similar, |E2A| was elevated in G−LVH+ compared with G+LVH+ patients (*P*=0.011 before correction for multiple comparisons). Differences remained after adjustment for fibrosis and hypertrophy (β=0.35 [95% CI, 0.15–0.55]; *P*<0.001; Figure 3A; Table 2).

#### Subclinical HCM (G+LVH−) Versus HV

Compared with HVs, patients with G+LVH− had evidence of microstructural alteration (lower FA, higher MD, and higher |E2A|), although this was less severe compared with patients with overt HCM (Table 2). Differences remained after adjustment for fibrosis and hypertrophy (FA: β=−0.45 [95% CI, −0.28 to −0.62], *P*<0.001; MD: β=0.39 [95% CI, 0.19–0.56], *P*<0.001; |E2A|: β=0.35 [95% CI, 0.17–0.52], *P*<0.001).

### Relationships of Microstructural Indices With Myocardial Perfusion

#### Overt HCM (All LVH+)

All 3 DTI parameters were associated with stress MBF (all *P*<0.02), but when fibrosis and hypertrophy were accounted for, only |E2A| was independently associated (β=−0.30 [95% CI, −0.11 to −0.47]; *P*=0.002; Figure 2).

#### Subclinical HCM (G+LVH−)

##### Perfusion Defects

Patients with G+LVH− with (n=21) compared with those without (n=54) focal perfusion defects were similar in age (*P*=0.60), sex (*P*=0.46), MWT (*P*=0.43), and ECV (*P*=0.85) but had a higher prevalence of LGE (5 [7%] versus 1 [1%]; *P*=0.006), similar global stress MBF (*P*=0.053), lower global MPR (2.58 [2.31–3.05] versus 3.15 [2.62–3.91]; *P*=0.011), and no difference in electrocardiographic abnormalities (*P*=0.62; Figure 3C).

Patients with G+LVH− with defects had evidence of more severe microstructural alteration (lower FA: 0.30 [0.29–0.32] versus 0.32 [0.31–0.33], *P*=0.002; higher |E2A|: 53.4° [47.1–59.8] versus 46.1° [41.3–53.0], *P*=0.009; but similar MD: *P*=0.47).

##### Global Perfusion

MPR but not stress MBF was associated with FA and |E2A|, and relationships remained after adjustment for fibrosis and hypertrophy (FA: β=0.33 [95% CI, 0.08–0.57], *P*=0.01; and |E2A|: β=−0.32 [95% CI, −0.07 to −0.57]; *P*=0.013).

### Relationships of Microstructural Indices With Electrocardiographic Abnormalities

#### Overt Disease (All LVH+) ECG

##### Overall

Of patients with overt HCM, 84% (85/101) had an abnormal ECG. Abnormal ECG was associated with all 3 DTI parameters (all *P*<0.001), stress MBF, and MPR (both *P*<0.016). When accounting for fibrosis, hypertrophy, and stress MBF, independent predictors of abnormal ECG were FA (OR, 3.3 [95% CI, 1.3–8.3]; *P*=0.01) and |E2A| (OR, 2.7 [95% CI, 1.2–6.0]; *P*=0.015). Stress MBF was also an independent predictor (OR, 2.8 [95% CI, 1.2–6.4]; *P*=0.015 when adjusted for FA, fibrosis, and hypertrophy).

##### Individual Electrocardiographic Abnormalities

Regarding individual electrocardiographic abnormalities, 28% (28) had abnormal Q waves; 71% (72) had T-wave inversion; 49% (49) had ST-segment depression; and 50% (50) met LVH voltage criteria. Logistic regression analyses of DTI/perfusion parameters and individual ECG abnormalities are included in Supplemental Results.

#### Nonsustained Ventricular Tachycardia

Thirteen percent (13) of patients had NSVT. NSVT was associated with lower stress MBF (*P*=0.026), higher MWT (*P*=0.002), higher LGE mass (*P*<0.001), and higher ECV (*P*=0.023). No DTI parameter was associated with NSVT (FA: *P*=0.08). No DTI or quantitative perfusion parameter was independently predictive of NSVT.

#### Subclinical HCM (G+LVH−) ECG

##### Overall

Of patients with subclinical HCM, 34% (26) had an abnormal ECG. An abnormal ECG was associated with all 3 DTI parameters (all *P*<0.001) and MPR (*P*<0.003). When adjusted for MPR, fibrosis, and hypertrophy, all 3 DTI parameters remained independently predictive of abnormal ECG (FA: OR, 4.0 [95% CI, 1.7–9.1], *P*=0.001; |E2A|: OR, 2.8 [95% CI, 1.4–5.7], *P*=0.006; MD: OR, 4.8 [95% CI, 2.0–11.4], *P*<0.001; and MPR: OR, 2.2 [95% CI, 1.0–4.9], *P*=0.049 [FA included as a covariate]).

##### Individual Electrocardiographic Abnormalities

Regarding individual electrocardiographic abnormalities, 21% (16) had abnormal Q waves; 9% (7) had T-wave inversion; 12% (9) met voltage criteria for LVH; and no participant had ST-segment depression. When we considered associations with individual electrocardiographic abnormalities in isolation, only FA and MD were predictive of abnormal Q waves after adjustment for MPR, fibrosis, and hypertrophy (FA: OR, 5.2 [95% CI, 1.6–16.0], *P*=0.006; MD: OR, 2.3 [95% CI, 1–5.3], *P*=0.049). There were no independent predictors of T-wave inversion, ST-segment depression, or LVH criteria.

## DISCUSSION

In the era of cascade genetic screening and emerging novel therapy, detection of phenotype development in subclinical HCM is an emerging priority. Study findings show that changes in DTI and quantitative perfusion occur even in the absence of hypertrophy. DTI abnormalities in subclinical disease relate to electrocardiographic abnormalities and perfusion defects, showing the likely importance of disarray and MVD in phenotype development.

Recent evidence suggests that genotype-positive HCM and genotype-negative HCM are different in LV morphology and clinical outcomes.^1,16,17^ In overt disease with LVH, all genotype-positive patients have perfusion defects, with 18% of genotype-negative patients having none. DTI abnormalities were unexpectedly more marked if the patient was gene-negative (more elevated |E2A|). This suggests that changes in DTI and quantitative perfusion are sensitive to mutation status.

This study is both the largest in vivo cDTI study performed to date and the largest prospective study of subclinical HCM. The study used a validated DTI sequence and consecutive recruitment from genetics databases of 3 referral cardiomyopathy centers where phenocopies are routinely screened. The exact mechanisms behind how disarray and MVD are present, even without hypertrophy, in those with pathogenic sarcomeric mutations are still elusive.

### Microstructural Changes, MVD, and Overt HCM

According to several preclinical models, DTI abnormalities are likely related to microstructural alteration (myocyte disarray and abnormal sheetlet orientation), although further human histological validation is needed.^15,29,31^ In line with other studies, overt disease was characterized by low FA and high MD.^12–15,22^ Although others found an independent association of FA and MD with fibrosis, our study findings also demonstrate an independent relationship with hypertrophy, mirroring previous histological work.^12,22,32^ Our study is the first to systematically examine the presence of microstructural abnormalities, including disarray, in patients with G+LVH+ and those with G−LVH+. Genotype-negative HCM has been hypothesized to result in part from polygenic inheritance.^17^ |E2A| elevation reflects a hypercontracted microstructural state in systole with failure to reorientate in diastole.^12,14,15^ Although G−LVH+ has been associated with less severe outcomes and fibrosis, here, it unexpectedly is related to elevated |E2A|, suggesting a more severe microstructural phenotype. Conversely, although both patients with G−LVH+ and those with G+LVH+ displayed considerable MVD compared with HVs, all G+LVH+ patients had visual perfusion defects compared with 82% of G−LVH+ patients. In overt disease, perfusion defects are associated with abnormal blood pressure response to exercise and, in apical HCM, aneurysm formation.^33,34^ In a previous positron emission tomography study, global MBF was more impaired in patients with G+LVH+ compared with those with G−LVH+, and although this was not found here, our study also supports the hypothesis of a more direct deleterious impact of sarcomeric mutation on microvascular function.^35^

### Microstructural Changes and Subclinical HCM

Subclinical HCM was characterized by LVH and fibrosis parameters similar to health, and despite this, DTI demonstrated lower FA, suggestive of more myocyte disarray. Although historically, disarray has been described in HCM for several decades, its presence in phenotype evolution was less understood.^8^ Recent 3-dimensional histological analysis uncovered that disarray is detected ex vivo in fetal murine models without hypertrophy.^9^ Our previous work and other investigations have modeled early disease by examining DTI parameters of nonhypertrophied myocardium in overt disease; however, this has limitations, as remote remodeling (in nonhypertrophied segments) occurs by the time LVH is detected.^18,22,36^ Elevated |E2A| in patients with G+LVH− suggests that the hypercontracted microstructural state demonstrated in overt disease is also present before hypertrophy.^12–14^ Elevated |E2A| may have a role in the hyperdynamic function, diastolic dysfunction, and impaired myocardial mechanics found in this cohort.^18,37^ Overall, our findings support the hypothesis that altered myocardial microstructure is an early phenomenon in the disease pathophysiology, in keeping with animal model work.^9^

### Microstructural Changes and Perfusion Relationship

Twenty-eight percent of patients with subclinical HCM had perfusion defects, and these had evidence of low FA, suggestive of more disarray, and more elevated |E2A| than those without perfusion defects. This relationship also persisted into overt disease, with markers of MVD being independently associated with markers of microstructural integrity. A unifying explanation for the findings of abnormal microstructure, fibrosis, and abnormal microvasculature is the capillary:myocyte coupling hypothesis, whereby microvasculature and the matrix meshwork are abnormal even during organogenesis.^7,38^ In overt HCM, compressive forces of hypertrophied myocardium and LV outflow tract obstruction also worsen ischemia.^39^ An interrelationship of perfusion, disarray, and myocardial mechanics could explain the compounding of perfusion and microstructural changes with LVH and their continued association in overt disease.

### MVD, Microstructural Changes, and Pathological Electrocardiographic Findings

In line with other studies in subclinical HCM, pathological electrocardiographic findings occurred in 34% and were more common in overt disease at 84%.^20,40^ The study shows for the first time that microstructural alteration (including lower FA, suggestive of cardiomyocyte disarray) is independently associated with electrocardiographic abnormalities in subclinical disease. Prognostically, pathological electrocardiographic findings in subclinical HCM are associated with a 4-fold increased risk of progression to overt disease.^20^ Mechanisms linking disarray to arrhythmia susceptibility include disruption of gap junctions, alteration of longitudinal to transverse conduction velocity ratios, and provision of different pathways for conduction.^8^ A pioneering DTI study elicited FA of the thickest LV segment associated with NSVT; however, histological studies demonstrate large variations in disarray from segment to segment.^32^ This relationship was not replicated here when global FA was used, accounting for the low number of individuals with NSVT in this relatively low-risk cohort (implantable devices excluded).^22^ Others have hypothesized that ischemia results in scar, which, in turn, leads to reentry circuits.^6^ We found an independent association of MVD parameters with abnormal ECG, with MPR associating with electrocardiographic abnormalities in subclinical HCM, suggesting that the arrhythmogenic potential of MVD could be more complex than scar formation itself.^20^

### Limitations

DTI parameters are variably influenced by the presence of fibrosis and other factors and therefore are not direct measures of disarray. However, the best available techniques were used for measurement of the important confounders: fibrosis, perfusion, and hypertrophy. MBF using adenosine stress is also an indirect measure of MVD and does not physiologically represent exercise. We are unable to fully exclude coronary disease, as systematic invasive coronary imaging was not performed. HVs did not undergo genotyping or Holter monitoring. Subclinical genotype-negative disease was not assessed. As with all contemporaneous cDTI sequences, susceptibility artifact, particularly in the inferolateral wall, was commonly found, and patients with devices were excluded.

### Conclusions

Microstructural alteration and MVD occur in overt HCM and are different in G+ and G− patients; both occur even in the absence of hypertrophy in sarcomeric mutation carriers, in whom changes are associated with electrocardiographic abnormalities. Microstructural alteration and MVD are associated in both subclinical and overt disease, suggesting relationships in phenotype development. Measurable changes in myocardial microstructure and microvascular function are early-phenotype biomarkers in the emerging era of disease-modifying therapy.

## ARTICLE INFORMATION

### Acknowledgments

The authors acknowledge Dr Sam Coveney’s contribution to the DTI data analysis. The authors also thank Sandy Gardner, Peter Sutton, Joel Isaacs, Kim Le, Carol McGann, Kirsty-Ann Bladen, Gemma James, Debbie Lewis, Sarah Hudson, Aysha McCall, Jodee Cooper, Dan Roberts, Carl Roberts, Shane Palmer, Ryan Evans, Katia Menacho, Justine Greenhorn, Simin Varghese, Muddassar Rashid, Saira Joy, Catherine Mammen, Gizem Pasa, Vera Mitchell, Salma Mohammed, Russell Hall, Hibba Kurdi, George Thornton, Ben Dowsing, Aderonke Abiodun, Christian Nitsche, Jonathan Bennett, Jessica Artico, Hunain Shiwani, João Augusto, Kristopher D. Knott, Andreas Seraphim, Andrew Lin, Clement Lau, Robert Adam, Konstantinos Moschonas, Petros Syrris, and Alessandra M. Ardissino.

### Sources of Funding

Dr Joy is funded by a British Heart Foundation clinical research training fellowship (FS/CRTF/21/2469) and has received funding from a Barts Charity project grant (MRC0281). Dr Das has received funding from Heart Research UK (RG2668/18/20). Dr Lorenzini has received consultancy fees from Pfizer, UK. Dr Manisty receives funding directly and indirectly from the National Institute for Health and Care Research Biomedical Research Centres at University College London Hospitals and Barts Health NHS trusts. Dr Lambiase is funded from University College London/University College London Hospitals National Institutes of Health and Care Research Biomedical Research Centre and Barts Biomedical Research Centre and by educational grants from Abbott and Boston Scientific. Dr Teh acknowledges funding from the British Heart Foundation (PG/19/1/34076). Dr Schneider acknowledges grant funding from a Wellcome Trust investigator award (219536/Z/19/Z). Dr Dall’Armellina has received funding from a British Heart Foundation intermediate clinical research fellowship (FS/13/71/30378). Dr Moon receives funding directly and indirectly from the National Institutes of Health and Care Research Biomedical Research Centres at University College London Hospitals and Barts Health NHS trusts, is the chief executive officer of MyCardium AI Ltd, and has served on advisory boards for Sanofi and Genzyme. Dr Lopes is supported by a Medical Research Council UK Research and Innovation Clinical Academic Research Partnership award (MR/T005181/1).

### Disclosures

None.

### Supplemental Material

Supplemental Methods

Supplemental Results

Figure S1

Tables S1–S3

## Supplementary Material


