# Supplementary material for: Microstructural and Microvascular Phenotype of Sarcomere Mutation Carriers and Overt Hypertrophic Cardiomyopathy
Source: Circulation. 2023 Jul 18;148(10):808–18. doi: 10.1161/CIRCULATIONAHA.123.063835 (PMC10473031; doi:10.1161/CIRCULATIONAHA.123.063835)
Supplement: Supplementary file 1 [file cir-148-808-s001.pdf]

SUPPLEMENTARY METHODS

CMR ACQUISITION

All scans were performed on a single 3 Tesla scanner (Siemens Prisma equipped with Gadgetron (running on a local external server) at Chenies Mews Imaging Centre (UCL), QS Enterprises. Imaging acquired: standard anatomical transaxial dark blood (HASTE) stack, balanced steady state free precession (bSSFP) cine imaging: three long axes and a short axis stack (11-14 slices), 3 short axis pre & post-contrast T1 mapping (Modified Look Locker Inversion with same-day haematocrit for ECV). 3 long axes and a short axis stack (11-13 slices) of averaged, motion-corrected, bright blood single shot bSSFP late gadolinium enhancement (LGE) imaging were also acquired post-contrast. **Quantitative Perfusion**: Fully-automated quantitative vasodilator stress perfusion was performed using a validated dual sequence approach (10). In brief, adenosine was given intravenously at 140-210mcg/kg/min for a minimum of 4 minutes until a minimum HR increase of 15 beats per minute and symptoms suggested an adequate physiological stress response. Gadolinium-based contrast (Dotarem, Gadoteric Acid, Guerbet, UK) was then administered intravenously at 0.05mmol/kg. The same was repeated for rest imaging 7 minutes after adenosine administration. Overall 27 HV completed stress perfusion (1 had respiratory motion artefact therefore stress perfusion maps were uninterpretable), 74 subclinical HCM underwent successful stress perfusion (1 declined contrast, 1 developed claustrophobia prior to contrast, 1 had evidence of visual perfusion defects on raw perfusion images but automated quantitative perfusion maps did not reconstruct successfully). All 101 overt HCM underwent successful stress perfusion. **cDTI**: A second order motion compensated single shot spin-echo echo-planar imaging diffusion tensor imaging sequence was performed for 3 short axis slices at peak systole using previously described protocols (12,13). This was acquired free-breathing without respiratory navigation and has been validated both ex-vivo, in-vivo and shown to detect microstructural changes in HCM (12,13,29). Acquisition parameters are as follows: TE/TR 77 ms/3 RR intervals, field of view 320x121 mm^2, matrix size 138x52, in-plane resolution 2.3 x 2.3 mm^2, 8mm slice thickness, 8mm inter-slice gap, and partial Fourier= 7/8. Scout diffusion-weighted (DW) data were acquired with diffusion-weighting applied in three orthogonal directions to ensure data quality. Each full data set comprised b-values of 100 s/mm^2 (3 DW directions, 12 repetitions), and 450 s/mm^2 (30 DW directions, 6 repetitions). Cine imaging was used to define the time from R peak to maximum systole. The trigger delay was set at 30% of maximum systole (12).

IMAGE ANALYSIS

Automated AI algorithms for cine imaging previously found to be superior to human precision were used to segment epicardial and endocardial volumes at end-diastole and end-systole and maximum wall thickness with trabeculations and papillary muscle excluded from blood pool (24,25). Pixelwise quantification of LGE was defined as signal intensity 5 standard deviations above the mean of a manually selected region of interest of normal myocardium and expressed in absolute mass in grams, referred to in results as "LGE". T1 and T2 parametric map analysis and LGE analysis was performed using Circle CVI software (version 5.14, Calgary, Canada). Automated contour detection of endo- and epicardial borders with manual adjustment where required were performed using the software with ECV to apply the formula (ECV = 1-Hct*($\Delta R1_{myocardium}/\Delta R1_{blood}$) where $\Delta R1_{myocardium} = 1/\Delta$ ($T1_{myocardium}$ pre - post-contrast) and $\Delta R1_{blood} = 1/\Delta$ ($T1_{blood}$ pre -post contrast) with same-day haematocrit (Hct). Septal ECV was used for regression analyses and referred to as ECV. Automated in-line adenosine stress perfusion maps were acquired to obtain stress and rest myocardial blood flow (ml/g/min) per AHA segment and globally (3 slices) (28). Visual perfusion defects were assessed qualitatively by experienced operators (GJ & JM). cDTI analysis was performed according to previously described protocols, in brief, data processing was performed using custom-built Matlab software (University of Leeds, UK, Matlab: Mathworks, MA, USA) with each image quality controlled for mis-registration or artefacts. Averaged magnitude images were generated from registered repetitions and were used to derive diffusion tensors. Segments containing artefacts were omitted from analysis. Contouring was performed to avoid the blood pool to minimise the effects of partial voluming.

SUPPLEMENTARY RESULTS

MATCHED HV vs OVERT HCM

The overt HCM group were age sex and ethnicity matched to 24 healthy volunteers (11 overlapped with the younger HV group described above). This was to detect if any DTI differences with the older HCM group and health remained when accounting for age. All 24 HV underwent DTI contrast CMR but 3 did not have ECV mapping.

Compared to overt HCM, healthy volunteers were well matched for age, sex and ethnicity (Age: HCM: 57(47-62) vs 51(42-61) years p=0.32, Female Sex: 24(24%) vs 7(29%) p=0.62, White: 77(76%) vs 20 (83%) p=0.43. As expected, overt HCM had lower LVEDVI (74±13 vs 84±23 ml/m^2 p=0.009), greater LVMi, (86(74-114) vs 60(49-75)g/m^2 p<0.001), higher MWT (17.2(15.4-21.1) vs 10.3(9.0-11.2)mm p<0.001), higher EF (79(74-83) vs 69(65-72)% p<0.001), higher ECV (28.7(26.4-32.6) vs 25.6(23.9-27.8)% p<0.001) and a higher prevalence of LGE (95(94%) vs 5(21%) p<0.001).

Compared to age, sex and ethnicity matched healthy volunteers, overt HCM had lower FA (0.28(0.25 vs 0.30) vs 0.34(0.33-0.36) p<0.001), higher MD 1.57 (1.53, 1.62) vs 1.48(1.44,1.54) $10^{-3}mm^2/s$ p<0.001) and higher |E2A| (61.9(56.5, 65.8) vs 43.4(37.9, 47.3)° p<0.001). Differences remained when correcting for fibrosis and hypertrophy (FA: β=-0.46, 95% CI:-0.31, -0.69 p<0.001, MD β=0.26, 95% CI: 0.07, 0.50 p=0.011, |E2A| β=0.52, 95% CI: 0.32, 0.63 p<0.001).

DEMOGRAPHIC RELATIONSHIPS OF DTI PARAMETERS

Age

In pooled healthy volunteers (n=41), there was no association between DTI parameters and age (FA: p=0.71, MD: p=0.99 |E2A|: p=0.95). In **subclinical HCM**, there was no relationship with age (FA: p=0.22, MD: p=0.93, |E2A|: p=0.10). **In overt HCM**, there was a positive association between MD with age (β=0.22 p=0.027), but not FA (p=0.89), or |E2A| (p=0.11).

Sex

Pooled Healthy Volunteers: DTI parameters were not different between male and female sex (FA: p=0.27, MD: p=0.97, |E2A|: p=0.32) In **subclinical HCM**, there was no relationship with sex: (FA p=0.34, MD: p=0.43, |E2A|: p=0.064). **In overt HCM,** there was no DTI relationship with sex (FA p=0.35, MD p=0.26, |E2A| p=0.52).

Ethnicity

Pooled Healthy Volunteers: DTI parameters were not associated with ethnicity: (FA: p=0.6, MD: p=0.6, and |E2A|: p=0.75). **In subclinical HCM,** there was no relationship with ethnicity (FA: p=0.90, MD: p=0.90, |E2A|: p=0.50). In **overt HCM**, there was no relationship with ethnicity (FA: p=0.28, MD: p=0.57, |E2A|: p=0.08).

Body Mass Index

There was no association between any DTI parameter and BMI in pooled healthy volunteers (FA: p=0.53, MD: p=0.47, |E2A|: p=0.82), subclinical HCM (FA: p=0.18, MD: p=0.42, |E2A|: p=0.84) or overt HCM (FA: p=0.12, MD: p=0.58, |E2A|: p=0.38).

COMPARISON BETWEEN G+LVH- & G+LVH+ (Supplementary Table 2)

Compared to G+LVH-, G+LVH+ were older, had lower prevalence of female sex, lower EDV (both absolute and indexed), higher EF and more severe markers of LVH and fibrosis. G+LVH+ was characterised by more severe microvascular disease (lower stress MBF and MPR) and more severe microstructural alteration (lower FA, higher MD, higher |E2A|). Differences remained for FA and |E2A| but not MD after adjusting for MWT, LGE and ECV (FA: β=-0.34, 95% CI: -0.07, -0.52 p=0.012 and |E2A| β=0.42, 95% CI: 0.14,0.58 p=0.002).

GENE SPECIFIC ANALYSES

Subclinical HCM. There was no significant difference in DTI parameters between thick vs thin filament mutations (FA p=0.70, MD p=0.94, |E2A| p=0.67). There was no significant difference in DTI parameters between MYH7 and MYBPC3 (FA p=0.11, MD p=0.77, |E2A| p=0.98). **Overt HCM**: In overt disease, thin filament mutations (n=10) were associated with a more elevated absolute |E2A| (62.8 (60.7, 64.6) vs 58.7 (55.5, 62.9)° p=0.029), but there was no difference in FA (p=0.84) or MD (p=0.74). There was no significant difference in DTI parameters between MYH7 vs MYBPC3 (FA p=0.09, MD p=0.31, |E2A| p=0.24).

ASSOCIATIONS OF DIFFUSION TENSOR, FIBROSIS AND HYPERTROPHY PARAMETERS

Pooled HV (n=41): There was no relationship between any DTI parameter and ECV (FA: p=0.61, MD: p=0.60, |E2A| p=0.48) or MWT (FA: p=0.32, MD: p=0.23, |E2A| p=0.24). 12%(5) pooled HV had LGE and there was no association with any DTI parameter (FA: p=0.58, MD: p=0.46, |E2A|: p=0.88).

G+LVH- vs HV: Table 2: Compared to healthy volunteers, G+LVH- had evidence of microstructural alteration (lower FA, higher MD, higher |E2A|) although this was less severe when compared to overt HCM. Differences remained after adjusting for fibrosis and hypertrophy (FA: β=-0.45,95% CI: -0.28, -0.62 p<0.001, MD: β=0.39, 95% CI: 0.19, 0.56, p<0.001, |E2A|: β=0.35, 95% CI: 0.17, 0.52 p<0.001).

G+LVH- & markers of fibrosis and LVH:

LGE: There were significant associations with |E2A| but not MD or FA (|E2A|: β=0.27, p=0.016). There were no associations between any DTI parameter and ECV or MWT.

Overt HCM vs HV, Supplementary Table 1: Compared to healthy volunteers, overt HCM had evidence of microstructural alteration: lower FA suggestive of disarray, higher MD and higher |E2A| suggestive of a more hypercontracted sheetlet configuration). Differences remained when adjusting for fibrosis and hypertrophy. (FA: β=-0.52, 95% CI: -0.32,-0.68 p<0.001, MD: β=0.26 95% CI: 0.05, 0.45 p<0.015, |E2A|: β=0.64 95% CI: 0.48, 0.80, p<0.001).

Correlations of markers of fibrosis and LVH: LGE: There were significant associations with all 3 DTI parameters (FA: β=-0.54, p<0.001, MD: β=-0.47 p<0.001 |E2A| β=0.29, p=0.003). **ECV:** There were significant associations with FA and MD but not |E2A| (FA: β=-0.34, p<0.001, MD: β=0.27 p=0.008). **MWT:** There were significant associations with FA, MD and |E2A| (FA: β =-0.33, p<0.001, MD: β =0.33, p<0.001, |E2A|: β=0.34, p<0.001). **Multivariable:** There were no interaction relationships between predictor variables and all associations with DTI parameters. When including LGE, ECV and MWT as covariates, only LGE was independently predictive of FA and MD but not |E2A| (FA: β=-0.26, 95%CI: -0.02,-0.50 p=0.033, MD: β=0.26, 95%CI:0.03,0.50 p=0.028). Only MWT was independently predictive of |E2A| (β=0.36, 95%CI 0.15, 0.58 p=0.001). There were no independent relationships between DTI parameters and ECV.

RELATIONSHIPS OF MICROSTRUCTURAL INDICES WITH INDIVIDUAL ECG ABNORMALITIES IN OVERT HCM

Overt HCM: Q-waves were present in 28% (28). TWI was present in 71% (72). ST-Depression was present in 49% (49). 50% (50) met LVH voltage criteria. 34% (34) had LVH with ST depression, 43% (43) had LVH criteria with T-wave inversion. **Q-waves:** No DTI parameter or markers of perfusion, fibrosis or hypertrophy were related to the presence of Q-waves. **T-Wave Inversion (TWI)**: TWI was associated with FA and MD (both p<0.003), stress MBF and MPR (both p<0.033), MWT (p<0.001) and LGE (p<0.001). When including markers of perfusion, fibrosis and hypertrophy, no DTI parameter was independently predictive of TWI, however stress MBF was independently predictive (OR=2.6, 95% CI 1.4, 5.0 p=0.003, (FA included in the model)). **ST-Depression.** ST-depression was associated all three DTI parameters (all p<0.026), stress MBF and MPR (both p<0.029), MWT (p<0.001) and LGE (p<0.001). When including stress MBF, MWT, ECV and LGE as co-variates, MD (OR=2.3, 95% CI: 1.2, 4.2, p=0.008) and stress MBF were independently predictive (OR=2.0, 95% CI 1.1, 3.8 p=0.033). **LVH Voltage criteria:** LVH by voltage criteria was associated with |E2A| and FA (both p=0.007) and MWT (p<0.001). No DTI parameter or stress MBF was independently predictive.

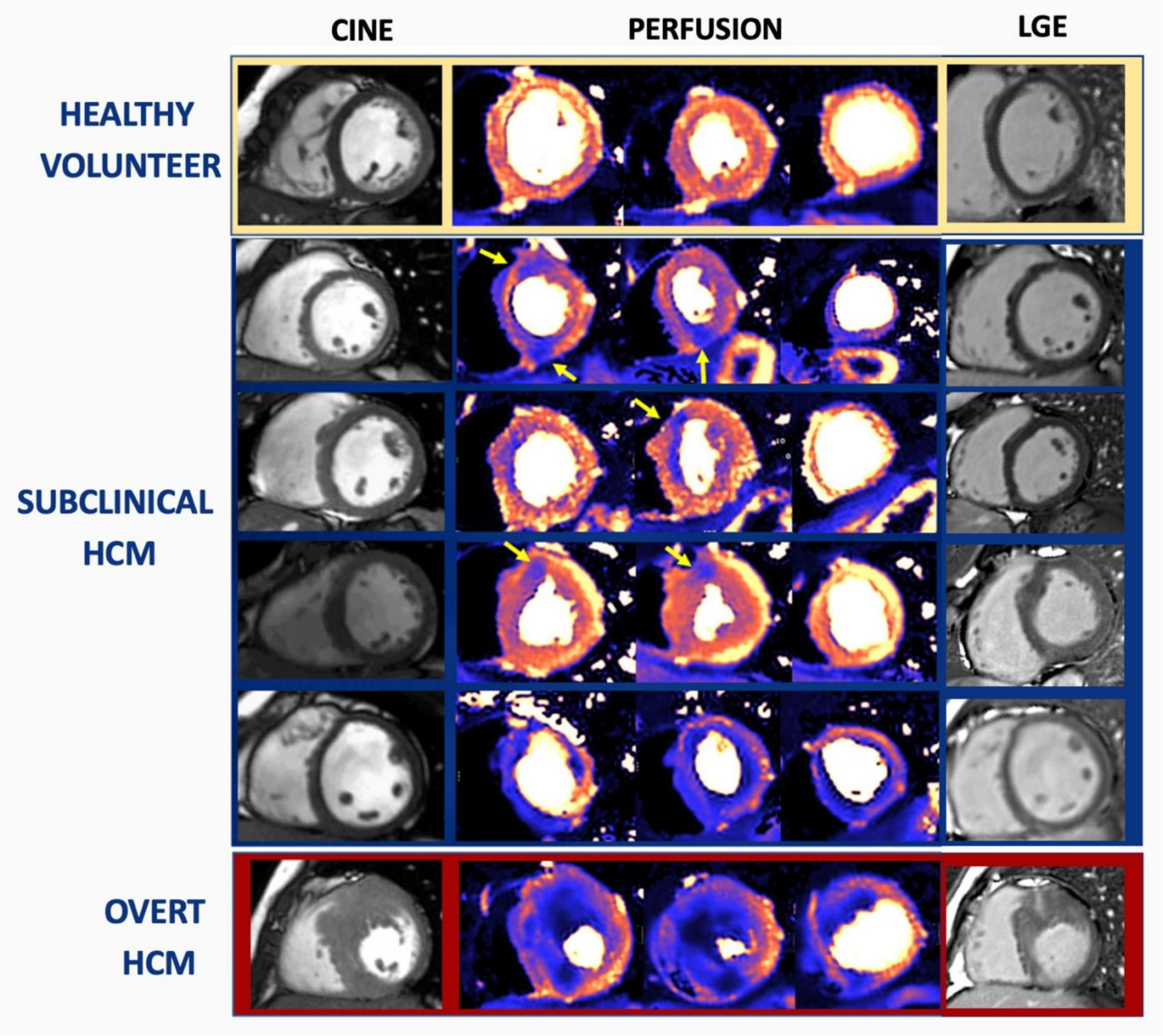

Supplementary Figure: Perfusion defects (indicated by yellow arrows) detected by quantitative perfusion as phenotype develops: subclinical HCM (sarcomere mutation carriers without hypertrophy): 28% prevalence of perfusion defect. Note the absence of scar (and when present, scar is minimal). In overt disease, ischaemia extends beyond scar (inducible ischaemia) and in G+LVH+, there is 100% prevalence. LGE – late gadolinium enhancement, HCM – hypertrophic cardiomyopathy

	Healthy Volunteers n=28	Overt HCM (All LVH+) n=101	p-value HV vs HCM
**Demographics**			
Age, years	34(32-39)	57(47-62)	**<0.001**
Female, n(%)	15(54)	24(24)	**0.002**
BSA, m^2	1.8±0.3	2.0±0.2	**0.009**
BMI (kg/m^2)	24(22-26)	25(24-28)	**0.013**
White, n(%)	20(71)	77(76)	0.89
Asian, n(%)	7(25)	18(18)	0.4
Black, n(%)	1(4)	6(6)	0.99
**Volumes & Mass**			
LVEDV index, ml/m^2	94±21	74±13	**<0.001**
LVEF, %	66(63-68)	79(74-83)	**<0.001**
MWT, mm	9.3(8.2-10.2)	17.2(15.4-21.1)	**<0.001**
LV Mass index g/m^2	53(45-68)	86(74-114)	**<0.001**
**Fibrosis markers**			
ECV septum, %	26.2(23.8-28.4)	28.7(26.4-32.6)	**<0.001**
LGE, present (%)	0	95(94%)	**<0.001**
LGE mass, g	0	7.1(3.0-15.1)	**<0.001**
**Microvascular disease**			
Stress MBF ml/g/min	2.77±0.62	1.69±0.51	**<0.001**
Rest MBF ml/g/min	0.77(0.68-0.86)	0.67(0.57-0.79)	**0.012**
MPR	3.47(2.90-3.75)	2.43(2.00-3.08)	**<0.001**
Visual perfusion defects n(%)	0	92/101(91%)	**<0.001**
**Diffusion Tensor**			
FA	0.34(0.33-0.36)	0.28(0.25-0.30)	**<0.001**
MD, $10^{-3}mm^2/s$	1.46(1.44-1.49)	1.57(1.53-1.61)	**<0.001**
|E2A|, °	41.6(34.9-47.2)	61.9(56.5-65.8)	**<0.001**

Supplementary Table 1: Demographics, volumetric and CMR parameters between Overt HCM and healthy volunteers.

	**Subclinical HCM (G+LVH-)** (G+LVH-) n=77		**Genotype Positive HCM** (G+LVH+) (n=51)	**p-value**
**Demographics**				
Age, years	31(23-40)	↑	52(37-59)	**<0.001**
Female n(%)	46(60)	↓	16(31)	**0.002**
BSA, m^2	1.9±0.2		1.9±0.2	0.1
BMI kg/m2	25(22-28)		25(24-28)	0.29
White, n(%)	64(83)		43(84)	0.95
**Volumes & Mass**				
LVEDV index, ml/m^2	83±14	↓	72±11	**<0.001**
LVEF, %	71(67-74)	↑	79(74-83)	**<0.001**
MWT, mm	9.6(8.6-10.6)	↑	17.2(15.4-21.1)	**<0.001**
LV Mass index g/m^2	51(43-60)	↑	77(67-92)	**<0.001**
**Tissue Characterization**				
ECV septum, %	27.2(25.3-29.7)	↑	30.0(27.1-33.8)	**<0.001**
LGE, present	6(8%)	↑	47(92%)	**<0.001**
LGE mass, g	0(range: 0-3.8)	↑	7.7(2.4-14.9)	**<0.001**
**Microvascular disease**				
Stress MBF ml/g/min	2.46±0.54	↓	1.77±0.52	**<0.001**
Rest MBF ml/g/min	0.80(0.66-0.93)	↓	0.69(0.59-0.83)	**0.036**
MPR	3.03(2.52-3.75)	↓	2.59(1.96-3.19)	**0.003**
Visual perfusion defects n(%)	21/75(28%)	↑	51(100)	**<0.001**
**Diffusion Tensor**				
FA	0.32(0.30-0.33)	↓	0.28(0.25-0.30)	**<0.001**
MD, $10^{-3}mm^2/s$	1.50(1.47-1.54)	↑	1.55(1.52-1.59)	**<0.001**
|E2A|, °	49.5(43.7-54.4)	↑	60.3(56.0-64.1)	**<0.001**

Supplementary Table 2 – comparison of Demographics and CMR, quantitative perfusion & diffusion tensor parameters between G+LVH- vs G+LVH+

PARTICIPANT NO.	SUBGROUP	GENE	NUCLEOTIDE SUBSTITUTION	AMINOACID SUBSTITUTION	ACMG (23) CLASSIFICATION
1	G+LVH-	TNNT2	304C>T	Arg102Trp	P
2	G+LVH-	MYH7	2167C>T	Arg723Cys	P
3	G+LVH-	MYH7	1207C>T	Arg403Trp	P
4	G+LVH-	MYBPC3	1405C>T	Gln469Ter	P
5	G+LVH-	MYBPC3	3163A>T	Lys1055*	LP
6	G+LVH-	MYBPC3	3330+5G>C	N/A	P
7	G+LVH-	MYBPC3	1898-23A>G	N/A	LP
8	G+LVH-	MYBPC3	1898-23A>G	N/A	LP
9	G+LVH-	MYBPC3	2373dupG	Trp792ValfsX41	P
10	G+LVH-	MYBPC3	2373dupG	Trp792ValfsX41	P
11	G+LVH-	MYBPC3	1504C>T	Arg502Trp	P
12	G+LVH-	MYBPC3	1227-13G>A	N/A	P
13	G+LVH-	MYBPC3	2827C>T	Arg943Ter	P
14	G+LVH-	MYBPC3	1624G>C	Glu542Gln	P
15	G+LVH-	MYBPC3	927-2A>G	N/A	LP
16	G+LVH-	TNNT2	304C>T	Arg102Trp	P
17	G+LVH-	MYBPC3	3491-2A>T	N/A	P
18	G+LVH-	MYBPC3	2371C>T	Gln791Ter	P
19	G+LVH-	MYBPC3	1224-19G>A	N/A	P
20	G+LVH-	MYBPC3	c.2950C>T	Gln984*	LP
21	G+LVH-	MYBPC3	305delCinsTGAGG	Pro102Leufs*12	LP
22	G+LVH-	MYBPC3	3476_3479dup	Pro1161Tyrfs*9	LP
23	G+LVH-	MYH7	2162G>A	Arg721Lys	P
24	G+LVH-	MYH7	1231G>A	Val411Ile	P
25	G+LVH-	MYBPC3	2864_2865delCT	Pro955Argfs*95	P
26	G+LVH-	MYH7	1988G>A	Arg663His	P
27	G+LVH-	MYBPC3	1624+4A>T	N/A	P
28	G+LVH-	MYBPC3	772G>A	Glu258Lys	P
29	G+LVH-	MYBPC3	1227-13G>A	N/A	P
30	G+LVH-	MYBPC3	927-2A>G	N/A	LP
31	G+LVH-	MYH7	1750G>A	Gly584Ser	P
32	G+LVH-	MYBPC3	1628delA	Lys543Argfs*12	LP
33	G+LVH-	TNNI3	433C>T	Arg145Trp	P
34	G+LVH-	TNNI3	470C>T	Ala157Val	LP
35	G+LVH-	MYBPC3	3330+5G>C	N/A	P
36	G+LVH-	MYH7	1207C>T	Arg403Trp	P
37	G+LVH-	TNNI3	433C>T	Arg145Trp	P
38	G+LVH-	MYH7	1816G>A	Val606Met	P
39	G+LVH-	TNNT2	304C>T	Arg102Trp	P
40	G+LVH-	MYL2	260G>C	Gly87Ala	LP
41	G+LVH-	MYBPC3	3181C>T	Gln1061*	P
42	G+LVH-	MYBPC3	2373dupG	Trp792ValfsX41	P
43	G+LVH-	MYH7	2389G>A	Ala797Thr	LP
44	G+LVH-	MYBPC3	1504C>T	Arg502Trp	P
45	G+LVH-	MYH7	2539A>G	Lys847Glu	P
46	G+LVH-	MYH7	2717A>G	Asp906Gly	P
47	G+LVH-	MYH7	1711G>A	Gly571Arg	LP
48	G+LVH-	MYBPC3	1504C>T	Arg502Trp	P
49	G+LVH-	MYH7	4259G>A	Arg1420Gln	LP
50	G+LVH-	MYBPC3	3163a>t	Lus1055*	P
51	G+LVH-	TNNT2	853C>T	Arg278Cys	LP
52	G+LVH-	MYBPC3	3163a>t	Lus1055*	P
53	G+LVH-	TNNI3	586G>A	Asp196Asn	P
54	G+LVH-	MYBPC3	3163a>t	Lus1055*	P
55	G+LVH-	MYBPC3	497delIT	Val166fs	P
56	G+LVH-	TNNI3	485G>A	Arg162Gln	P

57	G+LVH-	MYBPC3	3293G>A	Trp1098*	P
58	G+LVH-	MYH7	2162G>A	Arg721Lys	P
59	G+LVH-	MYBPC3	578A>G	Gln193Arg	P
60	G+LVH-	MYBPC3	2373dupG	Trp792ValfsX41	P
61	G+LVH-	MYH7	1063G>T	Ala355Ser	P
62	G+LVH-	MYH7	427C>T	Arg143Trp	P
63	G+LVH-	CSRP3	131T>C	Leu44Pro	LP
64	G+LVH-	MYBPC3	1484G>A	Arg495Gln	P
65	G+LVH-	MYH7	221G>C	Gly741Arg	P
66	G+LVH-	MYBPC3	1624G>C	Glu542Gln	P
67	G+LVH-	MYBPC3	2905+1G>A	N/A	P
68	G+LVH-	MYBPC3	1624G>C	Glu542Gln	P
69	G+LVH-	MYH7	2609G>A	Arg870His	P
70	G+LVH-	TNNT2	311C>T	Ala104Val	P
71	G+LVH-	TPM1	574G>A	Glu192Lys	P
72	G+LVH-	MYBPC3	772G>A	Glu258Lys	P
73	G+LVH-	MYBPC3	655G>C	Val219Leu	P
74	G+LVH-	MYBPC3	821+3G>T	N/A	LP
75	G+LVH-	MYBPC3	1504C>T	Arg502Trp	P
76	G+LVH-	MYBPC3	772G>A	Glu258Lys	P
77	G+LVH-	MYBPC3	Deletion exon 1 to 12	N/A	P

78	G+LVH+	MYBPC3	1224-52G>A	N/A	P
79	G+LVH+	TNNI3	c.485>C	Arg162Pro	P
80	G+LVH+	MYBPC3	1624+4A>T	N/A	P
81	G+LVH+	MYH7	427C>T	Arg143Trp	P
82	G+LVH+	MYH7	1207C>T	Arg403Trp	P
83	G+LVH+	MYH7	4130C>T	Thr1377Met	P
84	G+LVH+	MYBPC3	2373_2374insG	Trp792Valfs*41	P
85	G+LVH+	MYBPC3	2780_2781delCA	Thr927Ilefs*123	P
86	G+LVH+	MYBPC3	927-2A>G	N/A	LP
87	G+LVH+	TNNI3	470C>T	Ala157Val	P
88	G+LVH+	TNNI3	592C>G	Leu198Val	P
89	G+LVH+	MYH7	2080C>T	Arg694Cys	LP
90	G+LVH+	MYH7	2606G>A	Arg869His	P
91	G+LVH+	MYBPC3	1624G>C	Glu542Gln	P
92	G+LVH+	MYBPC3	2905+1G>A	N/A	P
93	G+LVH+	MYH7	2389G>A	Ala797Thr	P
94	G+LVH+	TNNT2	487_489delGAG	Glu163del	P
95	G+LVH+	TNNI3	407G>A	Arg136Gln	P
96	G+LVH+	MYBPC3	126G>A	Trp42*	LP
97	G+LVH+	MYBPC3	1236dup	Glu413Ter	P
98	G+LVH+	TNNC1	c.23C>T	Ala8Val	P
99	G+LVH+	MYH7	2302G>A	Gly768Arg	P
100	G+LVH+	MYBPC3	c.3697C>T	Gln1233*	P
101	G+LVH+	MYBPC3	1927+600C>T	N/A	LP
102	G+LVH+	MYBPC3	772G>A	Glu258Lys	P
103	G+LVH+	MYBPC3	1504C>T	Arg502Trp	P
104	G+LVH+	MYH7	4066G>A	Glu1356Lys	P
105	G+LVH+	MYH7	1063G>T	Ala355Ser	P
106	G+LVH+	MYH7	2681A>G	Glu894Gly	LP
107	G+LVH+	TNNT2	862C>T	Arg278Cys	P
108	G+LVH+	MYBPC3	1224-52G>A	N/A	P
109	G+LVH+	MYBPC3	2221G>C	Gly741Arg	P
110	G+LVH+	MYBPC3	1224-52G>A	N/A	P
111	G+LVH+	MYBPC3	2827C>T	Arg943Ter	P
112	G+LVH+	TNNT2	517G>A	Glu173Lys	P
113	G+LVH+	MYBPC3	1227-13G>A	N/A	LP
114	G+LVH+	MYH7	2389G>A	Ala797Thr	P
115	G+LVH+	MYBPC3	215delG	Gly72Alafs*24	LP

116	G+LVH+	TNNT2	275G>A	Arg92Gln	P
117	G+LVH+	MYBPC3	1224-19G>A	N/A	P
118	G+LVH+	MYBPC3	772G>A	Glu258Lys	P
119	G+LVH+	MYH7	1544T>C	Met515Thr	LP
120	G+LVH+	MYBPC3	1224-52G>A	N/A	P
121	G+LVH+	MYBPC3	3751T>C	Tyr1251His	LP
122	G+LVH+	MYBPC3	2373dupG	Trp792ValfsX41	P
123	G+LVH+	MYBPC3	747C>A	Cys249*	P
124	G+LVH+	CSRP3	449G>A	Cys150Tyr	LP
125	G+LVH+	MYBPC3	c.1504C>T	Arg502Trp	P
126	G+LVH+	MYH7	2609G>A	Arg870His	P
127	G+LVH+	MYBPC3	1224-52G>A	N/A	LP
128	G+LVH+	MYBPC3	1484G>A	Arg495Gln	LP

Supplementary Table 3: List of pathogenic/likely pathogenic variants in genotype positive participants, ACMG- American College of Medical Genetics
